# Satellite Glial Cells: Morphology, functional heterogeneity, and role in pain

**DOI:** 10.3389/fncel.2022.1019449

**Published:** 2022-10-06

**Authors:** Daria Andreeva, Lada Murashova, Nikita Burzak, Vyacheslav Dyachuk

**Affiliations:** Almazov Federal Medical Research Centre, Saint Petersburg, Russia

**Keywords:** satellite glial cells, chronic pain, pain conditions, nerve injury, heterogeneity

## Abstract

Neurons in the somatic, sympathetic, and parasympathetic ganglia are surrounded by envelopes consisting of satellite glial cells (SGCs). Recently, it has become clear that SGCs are highly altered after nerve injury, which influences neuronal excitability and, consequently, the development and maintenance of pain in different animal models of chronic pain. However, the exact mechanism underlying chronic pain is not fully understood yet because it is assumed that SGCs in different ganglia share many common peculiarities, making the process complex. Here, we review recent data on morphological and functional heterogeneity and changes in SGCs in various pain conditions and their role in response to injury. More research is required to decipher the role of SGCs in diseases, such as chronic pain, neuropathology, and neurodegenerative diseases.

## Introduction

The nervous system consists of two main cell types: neurons and glial cells. Glial cells have diverse functions in various physiological processes, including central nervous system (CNS) and peripheral nervous system (PNS) development (Pfrieger and Barres, 1997; Ullian et al., 2001; Christopherson et al., 2005; Pascual et al., 2005; Zuchero and Barres, 2015; Lago-Baldaia et al., 2020), pathogen recognition (Kofler and Wiley, 2011; Kigerl et al., 2014), cytotoxicity (Banati et al., 1993; Benn et al., 2001), extracellular matrix regulation (De Luca et al., 2020), lipid transport (Barber and Raben, 2019), cell-to-cell communication (Koizumi et al., 2005; Paolicelli et al., 2019; Schiera et al., 2019), and modulation of inflammation (Vallejo et al., 2010). Considering the great variety of peripheral glial functions, various mechanisms might play a role in different pain conditions, including the release of proinflammatory substances and neurotrophins (Suzumura et al., 2006; Vallejo et al., 2010; Mitterreiter et al., 2017), sensitizing neurons (Hossain et al., 2017).

The main types of glial cells in the CNS are astrocytes, microglia, oligodendrocytes, and ependymal cells, glial cells in the PNS include myelinating and non-myelinating Schwann cells, satellite glial cells (SGCs) and enteric glial cells. SGCs were functionally compared with astrocytes since astrocytes and SGCs, the main homeostatic glial cells, seem to share similar functions (Hanani and Verkhratsky, 2021). For many years, astrocytes have been a topic of great research interest compared to SGCs. Thus, comparing SGCs to astrocytes might be inaccurate and lead to imprecise impressions of SGCs functions and communication with neurons. Over the past few years, knowledge has been gained regarding the morphology, molecular heterogeneity, and involvement of SGCs in different pain conditions.

However, there is a lack of studies providing a comprehensive view of the recent advancements in our understanding of the SGCs. Therefore, this review aims to summarize the latest information about SGCs biology to advance our understanding of these glial cells.

## Morphological characteristics of satellite glial cells

The PNS is part of the nervous system that extends beyond the brain and spinal cord. It consists of cranial and spinal nerves, and plexuses of the autonomic nervous system. Cranial and spinal nerve bodies are located within the brainstem nuclei or in the dorsal root ganglia (DRG), while autonomic neurons are organized in the sympathetic and parasympathetic ganglia. As mentioned above, SGCs are one of the main types of glial cells in the PNS, including Schwann and enteric glial cells (Peripheral Glial Cells, 2013). SGCs are located in the sensory and autonomic ganglia of the PNS and form a tight sheath around the neuronal soma (Pannese, 1981). In some areas, SGCs simply contact each other, whereas, in other areas, the lamellar extensions of each SGC may intertwine and overlap (Pannese, 2010).

**Figure 1:**
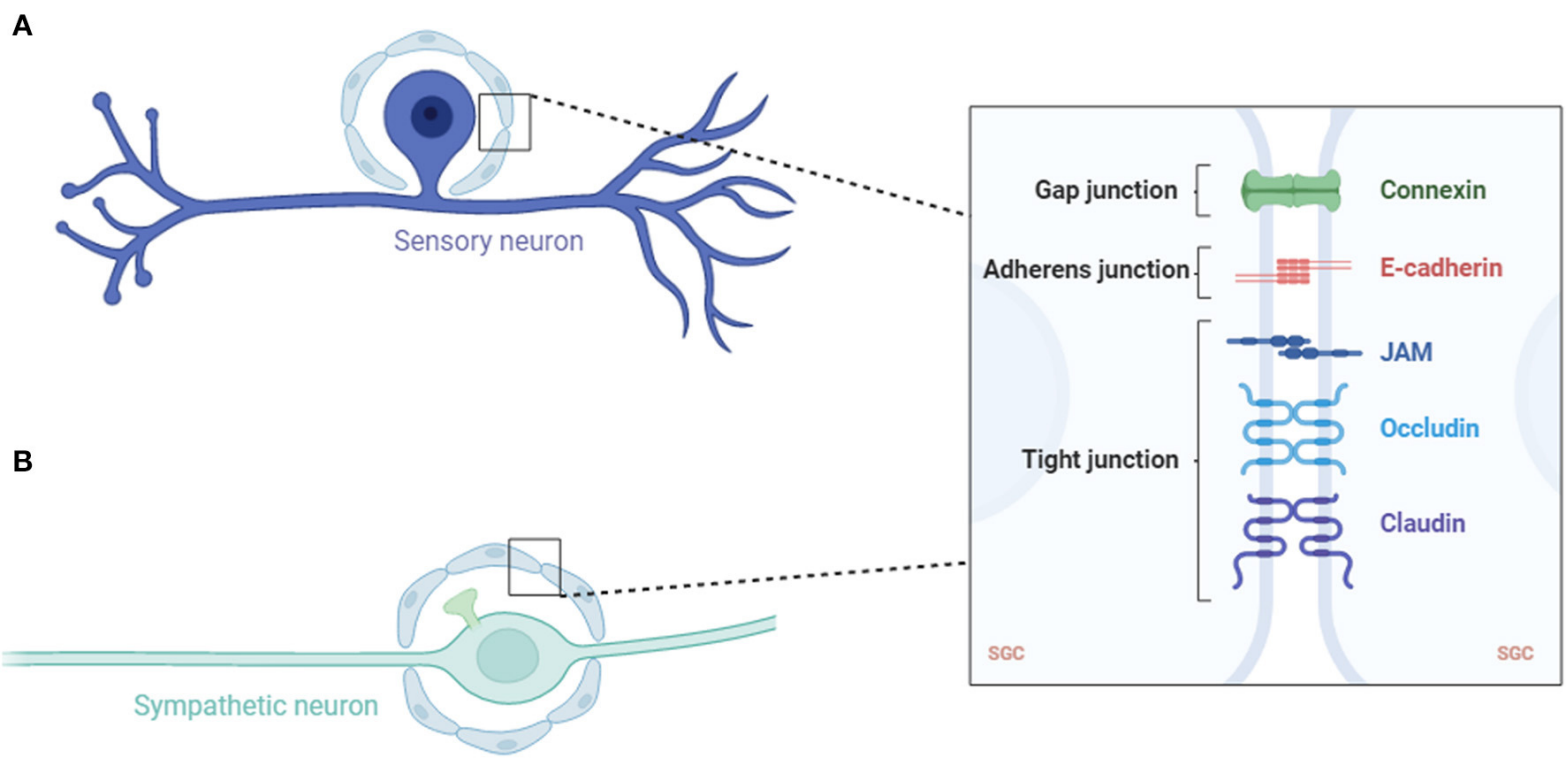
Schematic of a sensory **(A)** and sympathetic **(B)** neuron covered with an SGC envelope. SGC, satellite glial cell; JAM, junctional adhesion molecule.

The sensory ganglia, being DRG, trigeminal ganglia and other ganglia associated with cranial nerves, contain sensory neurons, SGCs, Schwann cells and other non-neuronal cells such as endothelial cells and immune cells (Haberberger et al., 2019; Vermeiren et al., 2020). SGCs in the sensory ganglia are laminar cells, usually a sheath of several SGCs that surround each neuron (Figure 1). The number of SGCs that make up the sheath increases in proportion to the surrounding neurons' volume (Hanani, 2005). Additionally, the envelope volume increases in proportion to the volume and surface area of the neuron. The distance of the extracellular space between the sheath and plasma membrane of the neuron is 20 nm, which allows DRG neurons and their SGC sheets to form a single anatomical and functional unit (Pannese, 1981). The patches of connective tissue separated these individual units. However, some sensory neurons occupy the same place in the connective tissue and are therefore divided by two or three neurons, which are primarily in newborn or young animals (Pannese et al., 1991).

**Figure 2:**
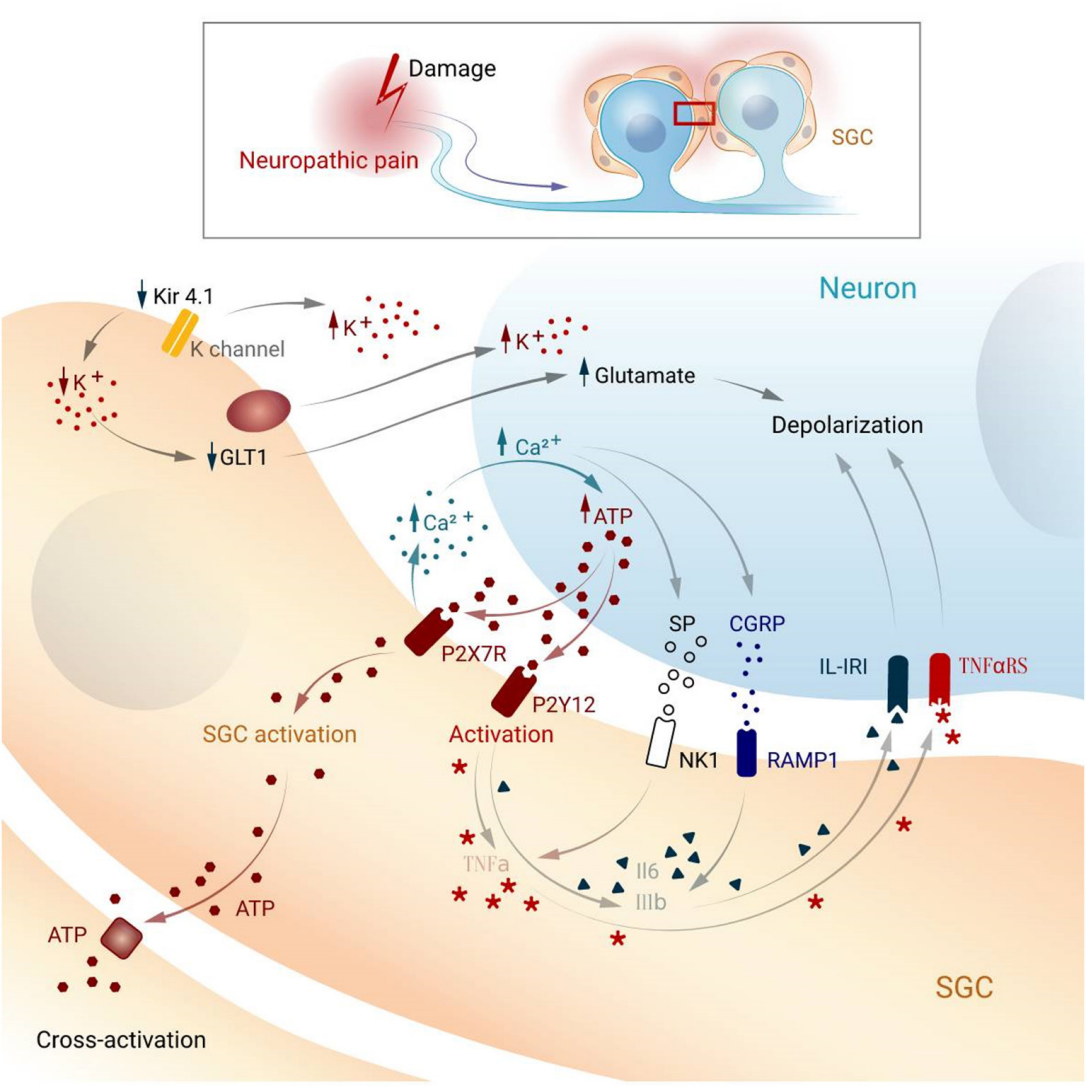
Possible mechanism underlying activation and cross-activation in SGCs and neurons while pain. SGC, satellite glial cell; Kir4.1, potassium channel; GLT1, glutamate transporter; P2X7R, P2X purinoceptor 7; P2Y12, purinergic receptor; NK1, Neurokinin 1; RAMP1, Receptor activity modifying protein 1; IL1-β, interleukin-1 beta; IK-IR, Interleukin-1 receptor; TNF-α, tumor necrosis factor-alpha; TNFaRS, tumor necrosis factor receptor; CGRP, Calcitonin gene-related peptide; SP, Substance P.

In the sympathetic ganglia, SGCs are one of the three main cell types, with the other two being sympathetic ganglion neurons and small highly fluorescent (SIF) cells (Hanani, 2010). The SIF cells of the sympathetic ganglia are divided into several groups, each surrounded by an SGC sheath. The SGCs of the sympathetic ganglia had the same basic structure as the sensory ganglia, except that the sympathetic ganglia also received synapses (Figure 2). Therefore, the SGC envelope of the sympathetic neurons must extend further to cover the axonal ganglia near the soma. Similar to the sheath area near the glial nucleus, axonal hillocks are thicker than those in the rest of the surrounding neurons (Hanani, 2010).

Much less knowledge has been gained about SGCs in the parasympathetic ganglia due to their location, which makes it difficult to access the ganglion. However, the general organization of the SGCs envelope is very similar to that in the sympathetic ganglia in guinea pig urinary bladder (Gabella, 1990), guinea pig trachea (Bałuk et al., 1985), guinea pig pancreas (Liu et al., 1997), mouse pulmonary vein ganglia (Bałuk and Gabella, 1987), mouse salivary duct ganglia (Pomeroy et al., 1996), cat pancreatic ganglia (Sha et al., 1996), human choroidal ganglia (May et al., 2004), and human cardiac ganglia (Pauziene and Pauza, 2003).

In all types of ganglia, SGCs have adhesive, tight, and gap junctions (Pannese et al., 1977, 1994; Sakuma et al., 2001; Liu et al., 2014). Molecules with a mass of up to 1 kDa can be transported through these gap junctions (Hanani et al., 2002; Pannese et al., 2003; Huang et al., 2006). This suggests that SGCs function in a synaptic context, thereby influencing the synaptic transmission.

Despite more than 50 years of studying SGCs morphology, our knowledge in this area is still limited. It is assumed that SGCs in different ganglia share many common peculiarities, therefore, more research dedicated to its complexity is needed.

## Heterogeneity of satellite glial cells

Sensory neurons in the DRG are extremely diverse depending on their size, expression, and signaling activity (Körner and Lampert, 2022). Because each neuron has a sheath consisting of SGCs, the diversity of neurons is accompanied by a variety of SGCs consisting of this sheath.

Studies using electron microscopy have demonstrated at least two morphological types of SGC (Siemionow et al., 2006; Nascimento et al., 2008): the first type (~50–60% of the population) is the most typical SGC, forming a sheath around the neuron with a very thin layer of cytoplasm and invaginating a lot inside the neuron. The second type consists of SGCs that are associated with an axon.

Modern molecular biology methods enable the isolation of cell subtypes based on analysis of their expression. Thus, RNA-seq analysis shows that SGCs exhibit a great variety in their molecular nature (Avraham et al., 2020; Tasdemir-Yilmaz et al., 2020; van Weperen et al., 2021; Mapps et al., 2022). The expression analysis of SGCs isolated from different locations showed specifically expressed various proteins. For example, SGCs of the cochlear ganglion share common markers (GATA 2, NPY, and Epha3) with other glial cells (Tasdemir-Yilmaz et al., 2020). Another study demonstrated a difference in protein expression of SGCs in the sensory and sympathetic ganglia (Mapps et al., 2022). The sensory subpopulation of SGCs is enriched in steroid biosynthesis and exclusively expresses Brevican core protein (Bcan), a member of the lectican family (Mapps et al., 2022). In addition, in both the DRG and sympathetic ganglia, there are three common types of SGCs: subpopulations enriched (1) for cytokine and interleukin signaling, (2) for ECM and cell adhesion pathways, and (3) for classical immediate early genes (Avraham et al., 2020; Tasdemir-Yilmaz et al., 2020; van Weperen et al., 2021; Mapps et al., 2022) also identified a specific SGCs subpopulation after injury enriched with (Prada et al., 2011) transcriptional factor, which regulates gliosecretion in astrocytes (Prada et al., 2011).

Thus, it is possible to distinguish several SGCs populations both within and outside the same ganglion: three populations that occur in each ganglion: SGCs expressing (1) proinflammatory molecules, (2) ECM and cell adhesion molecules, and (3) early genes, as well as a population that differs from ganglion to ganglion: (4) sympathetic SGCs and (5) sensory SGCs. Further study of the parasympathetic and other sympathetic and sensory ganglia SGCs will improve our understanding of the variety in morphological and molecular properties of SGCs, which is sufficient to understand their function in normal and pathological conditions, such as chronic pain, neuropathology, and neurodegenerative diseases.

## Participation of satellite glial cells in pain conditions

Pain is an unpleasant sensory experience associated with injury and/or damage. Pain is mainly mediated by nociceptors, the body of which lies in the DRG or trigeminal ganglia (Treede et al., 2019). Nociceptors are a unique neuronal population characterized by a high threshold of activation and unencapsulated nerve endings (Mertens et al., 2015). Nociceptive receptors are multimodal, non-myelinated, or lightly myelinated primary afferent nerve fibers. Their transmission is mainly mediated by glutamate, which modulates postsynaptic ionotropic receptors, which in turn can be modulated by the co-expression of substance P and calcitonin gene-related peptide (CGRP) (Zieglgänsberger, 2019). Pain plays a key role in the healing process, and consequently, in survival. People unable to experience pain rarely survive into adulthood, inevitably hurt themselves, and thus, decrease their life expectancy (Verpoorten et al., 2006).

Historically, pain has been divided into two main categories, representing the clinical aspects of pain syndrome (Bennett, 2006). Neuropathic pain is mainly caused by peripheral nerve injury, which leads to hyperalgesia due to enhanced sensory neuron excitability and reduced neuronal excitation threshold (Campbell and Meyer, 2006; Colloca et al., 2017). Inflammatory pain is usually considered to be an acute condition linked to nociceptor excitation and strong neuroimmune interactions that occur in response to tissue damage (Kidd and Urban, 2001).

Recent studies have identified the effects of neuropathic pain on SGCs (Ohara et al., 2008; Siemionow et al., 2009; Zhang et al., 2009; Ji et al., 2013; Yoon et al., 2013; Lee and Kim, 2020; Yuan et al., 2020). After nerve injury, SGCs may play an important role in the transmission of spinal cord injury signals (Ji et al., 2013). Studies have shown that gap junctions between glial cells may play an important role in neuropathic pain (Yoon et al., 2013). After nerve injury, the number of gap junctions between SGCs increases significantly (Ohara et al., 2008) but gradually returns to normal levels after a period of time. In models of neuropathic conditions, the number of activated SGCs connected with SGCs of other neurons is increased (Hanani et al., 2002; Lee and Kim, 2020; Yuan et al., 2020), while under physiological conditions, only a few neurons share a common sheath.

SGC activation is traditionally associated with increasing levels of glial fibrillary acidic protein (GFAP) (Siemionow et al., 2009; Zhang et al., 2009) and many other molecular properties. SGCs activation caused by increased GFAP expression leads to enhanced glial cohesion, whereas blocking gap junctions induces analgesia (Warwick and Hanani, 2013).

In the DRG, a large number of ions exist between cells to maintain the stability of cell potential, and satellite glia in the ganglion play an essential role in potassium ion buffering (Tang et al., 2010). Intracellular potassium homeostasis maintains neuronal excitability, which increases when the extracellular potassium concentration increases (Bellot-Saez et al., 2017). SGCs express the inwardly rectifying potassium channel Kir4.1, which buffers potassium concentrations in the ganglia (Tang et al., 2010). Studies have shown that Kir4.1 expression is downregulated upon nerve injury, and siRNA silencing of Kir4.1 induces spontaneous and evoked facial pain-like behaviors in freely moving rats (Ohara et al., 2008).

Glutamate is an important excitatory neurotransmitter in the CNS and PNS. Excessive glutamate can lead to increased neuronal excitation, which can lead to pain (Pereira and Goudet, 2019). The PNS does not contain glutamate-degrading enzymes; therefore, glutamate removal relies on high-affinity glutamate transporters on SGCs. SGCs take up extracellular glutamate and maintain homeostasis of extracellular glutamate (Fonseca et al., 2005; Chiang et al., 2007, 2008). The glutamate transporter, GLT-1, is involved in the transport of glutamate into glial cells (Maeda et al., 2008; Zhao et al., 2018). Following nerve injury, decreasing of Kir4.1 channel and sequential an increase of extracellular K+ can downregulate GLT-1 (Vit et al., 2008), accumulating glutamate, which increases the excitability of the postsynaptic neurons (Sung et al., 2003). Studies have shown that, in the pathological pain model, the glutamate content in the cell bodies of sensory neurons is increased (Kung et al., 2013; Cho et al., 2021). Additionally, SGCs also express glutamate-aspartate receptors and glutamine synthetase, so glutamate can be taken up outside the cell and converted into glutamine inside the cell. Glutamate uptake by SGCs can maintain normal extracellular glutamate levels, thereby maintaining neuronal excitability (Ohara et al., 2009).

After nerve injury, a large number of inflammatory cells at the injury site aggregate and release inflammatory transmitters, which induce chemical signals to generate electrical signals and transmit them to the DRG or trigeminal ganglion. Studies have shown that Adenosine triphosphate (ATP) is one of the main signal transmitters involved in communication between neurons and satellite glial cells (Hanani, 2012). While ATP cannot pass through the membrane, it is released by vesicles or channels such as P2X7R or P2Y12. When nerve impulses reach the DRG, SGCs and neurons release a large quantity of ATP, thereby increasing intracellular calcium concentration (Weick et al., 2003; Zhang et al., 2007; Suadicani et al., 2010; Villa et al., 2010). Activation of the purinergic receptor P2Y12R increases calcium influx into SGCs, which in turn increases cell excitability (Ceruti et al., 2008; Takeda et al., 2009; Katagiri et al., 2012). On the other hand, P2X7R is selectively expressed in SGCs and is involved in the modulation of nociceptive signals in the DRGs (North, 2002; Liu and Salter, 2005; Nakatsuka and Jianguo, 2006; Chen et al., 2008). For instance, P2X7R in SGCs promotes the release of proinflammatory cytokines including tumor necrosis factor-alpha (TNF-α), interleukin-1 beta, and interleukin-6 (IL-6) (Arulkumaran et al., 2011). In HIV treatment-induced neuropathic models, SGCs demonstrate increased GFAP expression and P2Y212 receptor activation (Zhou et al., 2019).

Transient receptor potential (TRP) channels are a group of ion channels that mediates sensory transduction. TRP type A1 (TRPA1) modulates calcium homeostasis in astrocytes (Shigetomi et al., 2011, 2013). A recent study revealed that inflammation and nerve injury enhance the expression of TRPA1 in neurons and SGCs, disrupting intracellular calcium signaling and leading to pain generation (Shin et al., 2020).

After nerve injury, electrical signals are transmitted into the ganglia, resulting in the massive release of neurotransmitters, as well as neural and immune factors such as glutamate, ATP, substance P, CGRP, brain-derived neurotrophic factor, IL-6, and CCL2 (Scholz and Woolf, 2007; Ren and Dubner, 2008; Milligan and Watkins, 2009). These mediators increase the sensitivity of postsynaptic neurons and activate satellite glia around the neurons. Peripherally released immune factors such as proinflammatory cytokines (e.g., IL-6) may also activate central glial cells (Schöbitz et al., 1992; Vallières and Rivest, 1997). Peripheral IL-6 can be transported to the CNS through the circulation, increasing COX-2 activity and PGE2 release in cerebral vascular endothelial cells, resulting in a central immune response (Schöbitz et al., 1992; Vallières and Rivest, 1997).

Glial cell activation and neuron-glia interactions play key roles in chronic pain. Accumulated data associate pain syndromes with various states of glial activation, occurring in SGCs as well as: glial response through upregulation of glial markers (i.e., GFAP), activation of ATP and glutamate transporters, and expression of glial mediators (e.g., cytokines, chemokines, growth factors). In this review, we report on recent developments in the involvement of SGCs in pathological conditions; however, our understanding is far from complete.

## Acute and chronic pain conditions

Acute and chronic pain are another category for pain. An acute pain condition has a brief onset and lasts <3 months, whereas chronic pain lasts longer than normal healing process. Acute pain characterizes a variety of inflammatory mediators expression (ATP, bradykinin, sodium, potassium, histamine and serotonin and others (Feizerfan and Sheh, 2015). These substances interact with cells surrounding injured cells, leading to depolarization and systemic inflammation, inducing up-regulation of P substances, activation TRP vanilloid receptors (TRPV) and, thus, to hyperalgesia (Pe et al., 2010). Repetitive stimulation may result a prolonged inflammation and release of different cytokines (such as IL-6, TNF-α and others), up-regulation of voltage-gated sodium channels, phosphorilation of protein kinases A and C. As described above SGCs involve in both acute and chronic conditions through different cellular mechanisms.

## Conclusion

Here, we describe the latest evidence on SGCs morphology, heterogeneity, and its role in various pain conditions. Despite the apparent importance of SGCs in normal and pathological conditions, our knowledge of their cell biology is still incomplete. Understanding SGCs biology might be indispensable to improving our understanding of chronic pain and other neurodegenerative diseases.

## Author contributions

DA and VD planned the study. AD, BD, LM, and NB wrote the manuscript. All authors read and approved the final version of the manuscript.

## Funding

This work was financially supported by the Ministry of Science and Higher Education of the Russian Federation (Agreement No. 075-15-2022-301). Open access funding was provided by the Almazov Federal Medical Research Center (Saint Petersburg, Russia).

## Conflict of interest

The authors declare that the research was conducted in the absence of any commercial or financial relationships that could be construed as a potential conflict of interest.

## Publisher's note

All claims expressed in this article are solely those of the authors and do not necessarily represent those of their affiliated organizations, or those of the publisher, the editors and the reviewers. Any product that may be evaluated in this article, or claim that may be made by its manufacturer, is not guaranteed or endorsed by the publisher.
